# Feeding behaviour of *Culicoides* spp. (Diptera: Ceratopogonidae) on cattle and sheep in northeast Germany

**DOI:** 10.1186/1756-3305-7-34

**Published:** 2014-01-18

**Authors:** Tania Ayllón, Ard M Nijhof, Wiebke Weiher, Burkhard Bauer, Xavier Allène, Peter-Henning Clausen

**Affiliations:** 1 Institute for Parasitology and Tropical Veterinary Medicine, Freie Universität Berlin, Robert-von-Ostertag-Str. 7-13, D 14163 Berlin, Germany; 2 CIRAD, UMR 15 Contrôle des maladies animales exotiques et émergentes, F 34398 Montpellier, France

**Keywords:** *Culicoides* species, Activity patterns, Preferential feeding sites, Host preferences, Cattle, Sheep

## Abstract

**Background:**

*Culicoides* spp. play an important role in the transmission of several vector-borne pathogens such as Bluetongue and Schmallenberg virus in Europe. To better understand the biology of local *Culicoides* species, a study divided into three parts was performed in northeast Germany to elucidate the feeding activity patterns (study A), preferential landing and feeding sites (study B) and host feeding preferences (study C) of *Culicoides* spp. using cattle and sheep as baits.

**Methods:**

In study A, the activity of *Culicoides* spp. was monitored over a 72 h period by collecting insects at regular intervals from the interior of drop traps with cattle or sheep standing inside. In study B, *Culicoides* spp. were directly aspirated from the coat and fleece of cattle and sheep during the peak activity period of *Culicoides*. In study C, *Culicoides* spp. were collected using drop traps with either cattle or sheep standing inside and located 10 m apart.

**Results:**

In study A, 3,545 *Culicoides* midges belonging to 13 species were collected, peak activity was observed at sunset. In study B, 2,024 *Culicoides* midges were collected. A significantly higher number of midges was collected from the belly and flank of cattle in comparison to their head region. In study C, 3,710 *Culicoides* midges were collected; 3,077 (83%) originated from cattle and 633 (17%) from sheep. Nearly half (46.7%) of the midges collected from cattle were engorged, significantly more than the number of engorged midges collected from sheep (7.5%). *Culicoides* from the Obsoletus complex (*C. obsoletus* and *C. scoticus*) were the most common *Culicoides* species encountered, followed by *C. punctatus*. Other species identified were *C. dewulfi*, *C. chiopterus*, *C. pulicaris*, *C. lupicaris*, *C. pallidicornis*, *C. subfascipennis*, *C. achrayi*, *C. stigma*, *C. griseidorsum* and *C. subfagineus*, the last two species are reported for the first time in Germany*.* Engorged *C. chiopterus* were collected in relatively high numbers from sheep, suggesting that this species may have a preference for sheep.

**Conclusions:**

An insight into the feeding behaviour of local *Culicoides* species under field conditions in northeast Germany was obtained, with implications for the implementation of control measures and midge-borne disease risk analysis.

## Background

Biting midges of the genus *Culicoides* are minute haematophagous flies with veterinary and medical importance as they can serve as vectors of several viruses, protozoa and nematodes. This includes Bluetongue virus (BTV), African horse sickness virus and Schmallenberg virus (SV) [1,2]. In recent years, the emergence of SV and the spread of BTV in Europe beyond its traditional geographic range exposed gaps in our understanding of factors which determine the emergence and expansion of midge-borne viruses in Europe. Bluetongue disease (BT) was considered an exotic disease restricted to southern areas of Europe, before its spread from 1998 throughout many countries in the Mediterranean Basin. In 2006, BT spread throughout Central and northern Europe where *C. imicola,* the major BTV vector in Africa and Southern Europe, was not found [3,4]. As a consequence, local Palearctic *Culicoides* species were suspected to be capable of transmitting BTV in northern Europe. Indeed, BTV was detected by PCR in native *Culicoides* species of the *Culicoides obsoletus* complex (which comprises *C. obsoletus sensu stricto* (ss) and *C. scoticus sensu stricto* (ss), as female *C. obsoletus* midges cannot be separated morphologically with certitude from *C. scoticus* midges) [5,6], *C. pulicaris*[7], *C. dewulfi*[8] and *C. chiopterus*[9]. Viral replication was demonstrated in *C. scoticus* following the artificial feeding of midges with BTV-spiked blood [10]. SV was detected in pools of biting midges from the Obsoletus group (comprising *C. obsoletus*, *C. scoticus, C. chiopterus* and *C. dewulfi*) [2,11]. Although the importance of autochthonous *Culicoides* species in the dissemination of BTV and SV is now generally recognized [3,12], many aspects of the ecology of native *Culicoides* species are still not fully understood, and there is a lack of information concerning their dispersal, vectorial capacity, feeding and host-seeking behaviour. The elucidation of the field biology of *Culicoides* midges is instrumental for the implementation of control measures and disease risk analysis, but is hindered by the small size of biting midges (1–3 mm).

The present work aimed to identify activity patterns, predilection sites and host feeding preferences for Palearctic *Culicoides* spp. feeding on cattle and sheep in the federal state of Brandenburg, northeast Germany. It was divided into three related studies. Study A aimed to monitor the daily activity pattern of *Culicoides* spp. in the vicinity of cattle and sheep. The objective of study B was to determine the predilection sites for landing and feeding of *Culicoides* species on cattle and sheep during periods of peak activity. Study C aimed to identify host feeding preferences of *Culicoides* species with cattle and sheep as animal baits, standing at 10 m intermediate distance. In studies A and C, midges present in the near proximity of sheep and cattle were collected using a drop trap method in combination with mechanical aspiration and in study B by direct mechanical aspiration alone, as these methods are considered to give more accurate representations of the midge biting rate in comparison to the use of UV light traps [13,14].

## Methods

### Study site

The pasture where the study was performed is located in the federal state of Brandenburg, Germany (52° 24′ 29.16“ N, 12° 46′ 17.39” E). Brandenburg is located in the transition zone between oceanic climate in Western Europe and continental climate in the East and has an average annual temperature of 9.8°C and annual rainfall of 611 mm (data 2003–2012) [15,16]. The pasture was largely surrounded by tall deciduous trees which provided a natural windbreak and shelter. Along the entire eastern and southern side of the pasture was an approx. 2 m wide drainage line with stagnant water providing a humid environment for *Culicoides* midges to breed. The suitability of the selected pasture to study *Culicoides* midges and other insects was ascertained in a previous entomological study performed in May-July 2012 during which large numbers of *Culicoides* species were collected using both UV light traps and the drop trap method [17].

For the present study, the pasture was divided into two evenly sized parts which were separated by electric fences and a 2 m wide corridor with three head of cattle on part A, and nine sheep on part B.

### Animals

Three Holstein Friesian heifers with an average body weight of 150 kg and 9 Merino sheep with an average body weight of approx. 50 kg were used for the study. All animals were in good health, had no noticeable body lesions and had not been previously treated with ectoparasiticides or macrocyclic lactones. Both animal groups were accustomed to electric fences. Animal health was checked and recorded during the entire study period, ethical approval for performing the study was obtained from the Ministry of Environment, Health and Consumer Protection, Potsdam. The metabolic weight (body weight ^0.75^) and body surface areas of heifers and sheep were calculated following the formulas of Brody (0.14 × body weight^0.57^) and Mitchell (0.09 x body weight ^0.67^) [18] respectively to determine group sizes that would minimize differences in basal metabolism and body surface areas between the different animal species. Following these calculations, it was decided to compare one heifer with three sheep in both study A and study C.

### Insect collection

**Figure 1:**
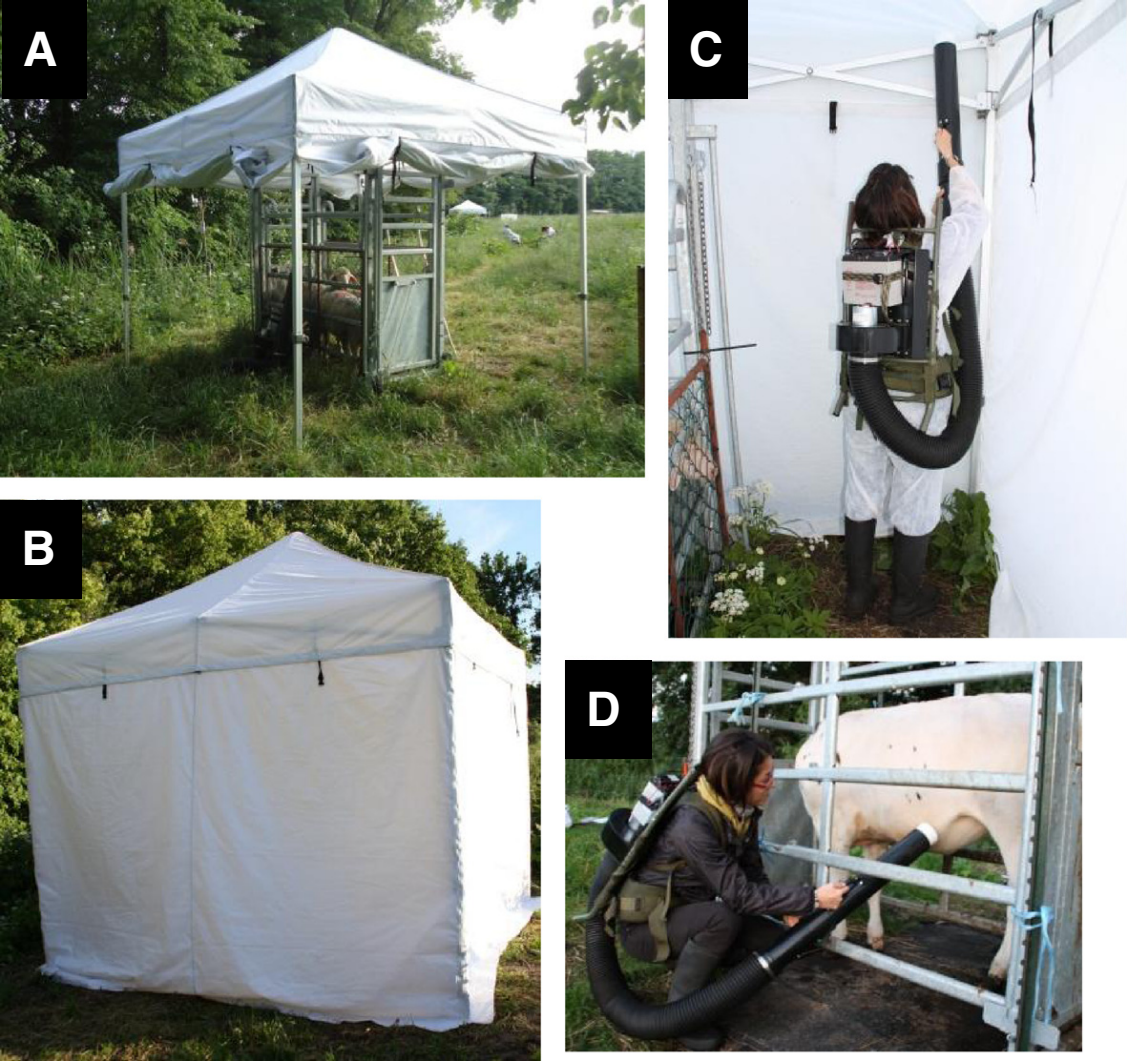
**Collection procedure performed during the study. (A)** drop trap and crush pen; **(B)** closed drop trap; **(C)** collection of midges by aspiration of the drop trap (study A and C); **(D)** collection of midges by direct aspiration (study B).

One crush pen was set up in each part, at approx. 2 m from the adjacent drainage line. Foldable marquees (Poptents, Aughnacloy, UK), hereinafter referred to as drop traps, with an aluminium frame, a peak height of 3.8 m and vertical walls of 3 m width and 2 m height (approx. inner volume 23.4 m^3^) were placed over each crush pen prior to each experiment in studies A and C (Figure 1). The drop traps were covered with white PVC lined polyester and the four side panels could readily be raised or dropped independently of each other. When the side walls were raised, insects could freely fly in and out of the drop trap, but insects in the near proximity of the animal standing inside the crush pen became trapped in the drop trap when the walls were dropped and zipped together on the sides. Insects were collected from the inside of the drop using a modified backpack aspirator to which a collection cup with a mesh size of 200 μm was attached (John W. Hock Company, Gainesville, FL). For the collections carried out in the dark, usually at 23:00 and 03:00 h, the investigating staff wore LED headlamps which were only lit inside the closed drop traps. After each collection, insects were stored inside the closed collection cups at 4°C, before being killed by storage at -20°C for at least 1 h. The insects were subsequently transferred to 15 mL Falcon tubes filled with 70% ethanol until identification. The investigating staff kept a minimum distance of >50 m from the drop trap during periods of non-activity. Since most insects do not fly during rainfall [19] and could be driven away or into the drop trap because of adverse wind conditions and temperature [1,20], the experiment was performed only under favourable weather conditions, i.e. collections were not performed during rainfall, when the temperature was under 10°C or when the wind speed was above 5 m/s. Weather data were recorded on a daily base in the direct vicinity of both crush pens using two wireless weather stations with a data logger (TFA Dostmann, Wertheim-Reicholzheim, Germany). During the entire study period, no other livestock grazed in the immediate surroundings (>300 m) of the study site.

### Experimental setting

#### Study A

The first study aimed to identify the activity pattern and feeding behaviour of *Culicoides* midges over 24 h, and was performed from 9–11 July 2012. During this period, insect collections from cattle and sheep were performed at defined time points (03:00, 04:00, 05:00, 06:00, 09:00, 12:00, 15:00, 18:00, 20:00, 21:00, 22:00 and 23:00 h) over three days. Collections were more frequently scheduled around sunset and dawn, which occurred at 21.27 and 04.56 hours, respectively, as most species of *Culicoides* are displaying crepuscular or nocturnal behaviour [1]. To limit any carry-over of insects from the cattle to the sheep group or *vice versa* by wind dispersal or active flight, an appropriate distance of approx. 120 m between the drop traps of the two animal groups was maintained. One heifer and a group of three sheep were transferred into the crush pens every 6–7 h and remained there for the collections. At the onset of every collection, the walls of the drop traps were raised for 15 min. The drop traps were subsequently closed for a period of 20 min, allowing the insects to contact the animals. After this period, the investigating staff quickly entered the drop trap and collected all insects from inside the closed drop trap using a backpack aspirator and collection cups for 15 min. After completion of the collection, the drop traps were re-opened for the second collection round, and this process was repeated at each of the time points given above. For welfare reasons, the animals standing inside the crush-pen were replaced by another animal or animal group from the pasture every 6–7 h.

#### Study B

Study B aimed to determine the predilection sites for feeding of *Culicoides* spp. on cattle and sheep. This study took place on the 17^th^, 18^th^, and 20^th^ July 2012. Four direct collections per day were performed by simultaneously aspirating the coat and fleece of one cow and one sheep for 10 min using the backpack aspirator, followed by a 15 min exposition interval between the collections. During the aspiration, the animals were standing inside their respective crush pens with 120 m intermediate distance. The study was performed during the peak activity period of the midges at sunset as determined in study A.

Four body regions were selected to identify preferential landing and feeding sites: back, belly/flank, head and legs. Back region comprised the withers to tail, going down to both sides until the half of the flanks; belly and flanks included the belly region and bottom half of the flanks; head comprised head and neck until the shoulders; legs covered from hoof to shank.

#### Study C

The last study was performed from the 23–25 July 2012, again during the peak activity of the midges at sunset (from 20:45 to 23:15 hours). Three collections were performed per day. The objective was to identify differences in host feeding preferences of *Culicoides* spp. In this case, the crush pen and drop trap from cattle was placed next to that of the sheep at an intermediate distance of 10 m. To control for the accidental carry-over of midges between both drop traps, the origin of the bloodmeal in all the engorged midges collected from sheep and the same number of randomly selected engorged midges from cattle was analysed by a PCR targeting the *cytochrome b* gene.

### Sample processing/ *Culicoides* identification

Collected *Culicoides* spp*.* were counted and identified to species level under a stereomicroscope (Carl Zeiss Microscopy GmbH, Göttingen, Germany), according to identification keys [21,22]. The *C. lupicaris*, *C. stigma* and *C. subfagineus* specimens could only be identified after dissection on slide preparations. Midges were examined and divided into males, engorged and non-engorged females. The parity status (parous-nulliparous) of non-engorged female midges was also recorded.

### DNA extraction and PCR

All 47 engorged midges collected from sheep in study C and an equal number of randomly selected engorged midges collected during the same study from cattle were individually homogenized in 1.5 mL Eppendorf tubes with a sterile microtube pestle (Dunn Labortechnik, Asbach, Germany) and their DNA was subsequently extracted using the NucleoSpin® Tissue kit (Macherey-Nagel, Düren, Germany) following the manufacturer’s instructions.

DNA-samples were subjected to bovine and ovine specific PCR-amplification targeting the cytochrome b (*cyt b*) gene as described by Garros *et al*. [23] with minor modifications. The amplification was performed using Maxima Hot Start Taq DNA polymerase (Thermo Scientific, St. Leon-Rot, Germany) according to the manufacturer’s protocol in a final reaction volume of 25 μL. Touch-down PCR conditions were as follows: one cycle at 95°C for 5 min, followed by 20 cycles at 95°C for 30 s, 65°C for 30 s and 72°C for 30 s, with a decrease of the annealing temperature of 0.5°C for each cycle, followed by 20 cycles at 95°C for 30 s, 55°C for 30 s and 72°C for 30 s, and a final elongation step of 72°C for 10 min. The specificity of the primers was confirmed by using each primer set with control DNA from sheep and cattle and unfed midges. Ten μL of each amplified products were visualized after gel electrophoresis on 1% agarose gel stained with GRGreen DNA Stain (Excellgen, Inc., Rockville, MD).

### Statistical analysis

All statistical analyses were performed using the IBM SPSS Statistics 20.0 program. The analyses aimed to compare the differences in the four body regions selected for the collections by direct aspiration, and to check whether there were statistically significant differences in the number of midges collected from cattle and sheep. Furthermore, with this analysis the possibility of an influence of the different animals used every day in the results was tested. A mixed ANOVA model for repeated measurements was used in the analysis, where cattle and sheep, which were different every day to minimize individual differences in host attractiveness, were considered as random effects. The four different body regions (head, back, belly/flanks and legs) and the two groups of animals (cattle and sheep) were considered as fixed effects in study B and study C respectively. The data (number of midges) was log transformed (n + 1) to limit the differences caused by outliers. Numbers of engorged and non-engorged females collected from cattle and sheep were compared using a chi-square analysis for studies A, B and C. The level of significance (p-value) was determined at 0.05.

## Results

### Study A: activity pattern over 24 hours

**Figure 2:**
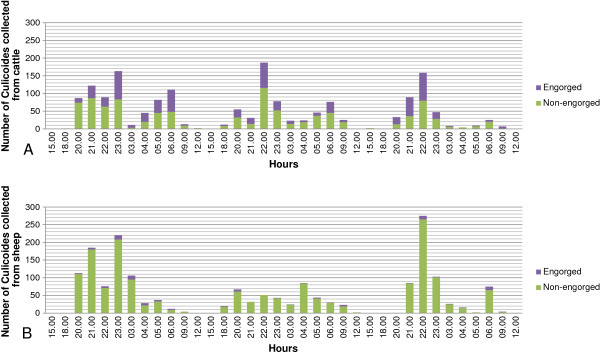
**Number of female *Culicoides *spp. collected from (A) cattle and (B) sheep in study A.** The females are classified according to their physiological status. In study A, the activity pattern of midges was determined by making 36 collections at defined time points over a 72 h period using two drop traps located approx. 120 m apart, with either one heifer or a group of three sheep standing inside. During the collection period, sunset occurred at 21.27 and sunrise at 04.56.

During the 36 collections performed from cattle and sheep over a period of three days, a total of 3,545 *Culicoides* (3,497 females and 48 males) belonging to 13 species were collected. Similar numbers of *Culicoides* were collected for cattle (n = 1,720) and sheep (n = 1,825). For all females for which the physiological status could be determined (n = 3,456), significantly more engorged midges were collected from cattle (n = 677, 40.6%) than from sheep (n = 95, 5.3%) (p < 0.001). Similar activity patterns were observed during all three days: the highest number of midges was collected around 22.00 (24% of the catches), and a smaller peak was observed around 06.00. Very few (n < 5) *Culicoides* biting midges were collected during daytime at the 12:00 and 15:00 collections (Figure 2).

The large majority of *Culicoides* collected from cattle and sheep belonged to the Obsoletus complex (*C. obsoletus* and *C. scoticus*), followed by *C. punctatus* and *C. pallidicornis*. Other species of *Culicoides* identified were *C. dewulfi*, *C. chiopterus*, *C. pulicaris*, *C. lupicaris*, *C. subfascipennis*, *C. achrayi*, *C. griseidorsum*, *C. subfagineus* or *C. stigma* (Tables 1 and 2). Some specimens (n = 119) were damaged and not classifiable at species level, and were categorized as *Culicoides* spp.

**Table 1:** **Female *Culicoides *** **species spectrum and number of specimens collected from cattle**

	**Study A**					**Study B**					**Study C**				
**Species**	**♀ P**	**♀ NP**	**♀ E**	**♀ NI**	**Total**	**♀ P**	**♀ NP**	**♀ E**	**♀ NI**	**Total**	**♀ P**	**♀ NP**	**♀ E**	**♀ NI**	**Total**
Obsoletus complex	284	414	530	18	1246	388	696	152	7	1243	324	869	1236	15	2444
*C. dewulfi*	1	-	2	-	3	2	2	-	-	4	-	1	-	-	1
*C. chiopterus*	2	-	1	-	3	11	3	3	-	17	59	7	40	-	106
*C. punctatus*	58	66	48	1	173	42	98	13	1	154	120	147	62	1	330
*C. pulicaris*	1	3	1	-	5	1	1	-	-	2	-	-	-	-	-
*C. lupicaris*	-	-	-	-	-	-	-	-	-	-	-	-	-	-	-
*C. pallidicornis*	36	49	47	-	132	53	114	36	-	203	36	34	71	3	144
*C. subfascipennis*	4	11	8	-	23	26	29	17	-	72	5	3	2	-	10
*C. achrayi*	7	6	2	-	15	-	-	-	-	-	4	3	3	-	10
*C. griseidorsum*	6	3	2	-	11	-	1	-	-	1	3	6	6	-	15
*C. subfagineus*	-	-	1	-	1	-	-	-	-	-	-	-	-	-	-
*C. stigma*	-	-	-	-	-	-	-	-	-	-	-	-	-	-	-
*Culicoides* spp.	18	19	35	14	86	-	-	-	-	-	4	3	4	-	11
TOTAL	417	571	677	33	1698	523	944	221	8	1696	555	1073	1424	19	3071

♀P: parous females; ♀NP: nulliparous females; ♀E: engorged females; ♀NI: females not identifiable by their physiological status due to damage.

**Table 2:** **Female *Culicoides *** **species spectrum and number of specimens collected from sheep**

	**Study A**					**Study B**					**Study C**				
**Species**	**♀ P**	**♀ NP**	**♀ E**	**♀ NI**	**Total**	**♀ P**	**♀ NP**	**♀ E**	**♀ NI**	**Total**	**♀ P**	**♀ NP**	**♀ E**	**♀ NI**	**Total**
Obsoletus complex	652	769	83	7	1511	89	161	7	1	258	89	172	21	1	283
*C. dewulfi*	3	-	2	-	5	-	-	-	-	-	-	-	-	-	-
*C. chiopterus*	4	-	-	-	4	3	-	-	-	3	69	2	23	1	95
*C. punctatus*	118	72	6	1	197	7	18	-	-	25	57	110	3	-	170
*C. pulicaris*	-	-	-	-	-	-	-	-	-	-	-	-	-	-	-
*C. lupicaris*	-	1	-	-	1	-	-	-	-	-	-	-	-	-	-
*C. pallidicornis*	12	14	2	-	28	9	20	-	-	29	29	34	-	-	63
*C. subfascipennis*	4	1	-	-	5	4	5	1	-	10	1	9	-	-	10
*C. achrayi*	3	7	-	-	10	-	-	-	-	-	4	5	-	-	9
*C. griseidorsum*	3	1	-	-	4	1	-	-	-	1	1	-	-	-	1
*C. subfagineus*	-	-	-	-	-	-	-	-	-	-	-	-	-	-	-
*C. stigma*	-	1	-	-	1	-	-	-	-	-	-	-	-	-	-
*Culicoides spp.*	17	14	2	-	33	-	-	-	-	-	-	-	-	-	-
TOTAL	816	880	95	8	1799	113	204	8	1	326	250	332	47	2	631

♀P: parous females; ♀NP: nulliparous females; ♀E: engorged females; ♀NI: females not identifiable by their physiological status due to damage.

### Study B: preferential feeding locations on the host

In this study, 2,023 midges (one male and 2,022 females) comprising 8 species were collected from cattle and sheep.

**Figure 3:**
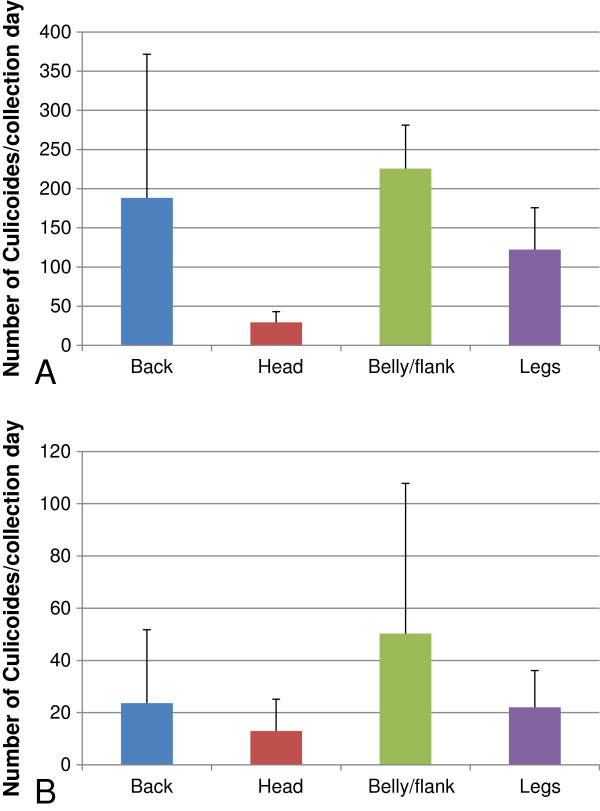
**Preferential landing and feeding sites of *Culicoides *midges on cattle and sheep.** Bars represent the mean number (± standard deviation) of *Culicoides* spp. which were aspirated from the coat and fleece of four selected body regions from **(A)** cattle and **(B)** sheep using a backpack aspirator over a 10 min period during the peak activity of midges at sunset.

By direct aspiration, a higher number of midges were collected from the belly and flank of cattle, followed by the back, but we also collected a high number of midges from the legs (Figure 3A). Significantly fewer midges were collected from the head in comparison to all other body regions (p < 0.001). From all female midges collected from cattle for which the physiological status could be determined (n = 1,688), 13.1% were engorged, 31.0% were classified as parous and 55.9% as nulliparous.

Although a higher number of midges was collected from the belly, flanks and legs of the sheep, no statistical differences were found between the four body regions (p = 0.139) (Figure 3B). Only 8 (2.4%) of the identifiable female midges (n = 325) collected were engorged, a difference which was statistically significant in comparison to the number of engorged midges collected from cattle (p < 0.001), and 35.0% and 62.6% were classified as parous and nulliparous, respectively.

The dominant species of *Culicoides* collected from both cattle and sheep in study B belonged to the Obsoletus complex (*C. obsoletus* and *C. scoticus*), followed by *C. pallidicornis* (Tables 1 and 2).

### Study C: feeding preferences for sheep and cattle

In study C, 3,710 *Culicoides* (n = 3,077, 83% from cattle, and n = 633, 17% from sheep) were collected, 3,702 of which were females and 8 males. A total of 46.7% of the female midges for which the physiological status could be determined collected from cattle were engorged, and 18.2% and 35.1% were classified as parous and nulliparous, respectively. Nineteen midges were not classifiable at this level (Table 1). In sheep, only 7.5% of the midges were engorged, 39.7% were parous and 52.8% nulliparous. In this case, two midges were not classifiable according to their feeding and parity status (Table 2). Statistically significant differences were observed between the number of collected midges between cattle and sheep (p = 0.008) and their feeding status (p < 0.001).

In both cattle and sheep, *Culicoides* from the Obsoletus complex (*C. obsoletus* and *C. scoticus*), were dominating, followed by *C. punctatus* and *C. pallidicornis* in cattle, and *C. punctatus* and *C. chiopterus* in sheep (Tables 1 and 2). Also identified were *C. dewulfi*, *C. pulicaris*, *C. subfascipennis*, *C. achrayi* and *C. griseidorsum*. The fraction of engorged *C. chiopterus* midges collected from sheep was significantly higher (p = 0.048) in comparison to that of cattle. Few *Culicoides* specimens (n = 11) were not identifiable at species level, and were classified as *Culicoides* spp.

The bloodmeal origin of all the engorged midges collected from sheep (n = 47) and the same number of randomly selected engorged midges from cattle was tested by PCR targeting the *cyt b* gene. From the 94 engorged midges analysed by PCR, 46 midges (97.9%) collected from sheep and 45 midges (95.7%) collected from cattle contained only DNA from sheep and cattle respectively. The three remaining midges, one collected from sheep and two collected from cattle tested negative for both ovine and bovine specific PCRs.

## Discussion

In study A, we observed a bimodal pattern of activity, with peaks at dawn and dusk. The highest midge activity was found around sunset, followed by a smaller peak of activity at dawn. This is in line with previous reports on the ecology of *Culicoides* in Europe [24,25]. Stabling of animals at dusk and dawn might curtail the risk of disease transmission as a result of a higher activity of midges during that time of the day. However, midges are also frequently found inside stables [13,26] and stabling of animals will therefore not sufficiently protect animals from midge bites.

*Culicoides* spp. were predominantly collected from lower body parts (belly and flank) from cattle and sheep in study B. These results are particularly important in relation to the pharmacokinetics of topical (pour-on) antiparasitic drugs, as their dispersal after administration on the backline was found to be inhomogeneous, with lower concentrations found on the ventral and distal regions [27,28]. This may limit the efficacy of pour on treatments against *Culicoides* biting midges [29].

Significantly more midges were collected from cattle in all studies, indicating that cattle may be more attractive than sheep for midges, as previously suggested by other authors [23,30]. This has important epidemiological implications, as sheep could be a susceptible target for these vectors when cattle are absent in the vicinity. In addition, significantly more engorged midges were collected from cattle in all the studies, contrasting with other studies, where a relative low number of fully engorged female midges were collected [30,31] which could possibly be explained by differences in collection methods. However, our results support the hypothesis that cattle not only attract midges but they also constitute important hosts.

Nearly all of the midges collected from cattle and sheep which were analysed by PCR, had fed on cattle and sheep, respectively. This confirms that *Culicoides* spp. which were collected on cattle or sheep, had indeed gorged themselves on the respective animals. These results underpin the suitability of drop traps as a collection method to evaluate the host feeding preferences of midges for different animal species. The validity of the employed methods is also supported by the low number of *Culicoides* males collected during this study, indicating that we caught mostly host-seeking females.

From the checklist of the 76 *Culicoides* spp. reported to occur in Germany [32], we collected and identified 11 species. Two other species (*C. subfagineus* and *C. griseidorsum*) are reported here for the first time in Germany. Time of day, trapping method, geographical region, meteorological conditions, seasonality and the proximity of other animals may influence the species composition of *Culicoides* collected [24]. Several methods of catching flying insects have been described [14,33-36]. Different trapping methods may suggest different activity patterns [37]. Certain collection methods such as light traps could impose a bias on the populations of *Culicoides* present in the area [13], we used a drop trap in combination with mechanical aspiration by a modified backpack aspirator. This method had been shown to catch more midges compared to other collecting methods [14]. In study B, we performed direct aspiration of midges without using a drop trap to examine the preferential landing and feeding sites on cattle and sheep. The number of collected engorged midges was comparatively lower in study B where direct aspiration from the animals was performed as opposed to studies A and C where the drop trap was used. One plausible explanation could be that some midges were collected by direct aspiration before they fed, whereas the drop trap allowed midges more time to feed.

*Culicoides* species from the Obsoletus complex (*C. obsoletus* and *C. scoticus*), *C. pulicaris*, *C. dewulfi* and *C. chiopterus* have been suspected to be BTV vectors in the absence of *C. imicola*[5,7,9,10,38] and some of them have also been incriminated as vectors in the transmission of SV [2,11]. During the whole period of the present work, *Culicoides* from the Obsoletus complex were the most common *Culicoides* species collected. This is in agreement to previous reports from Germany and other northern European countries, where this complex made up to 90% of all *Culicoides* midges in collections which were made following the BT outbreak of 2006 [5,13,39-42]. The high numbers of midges from the Obsoletus complex in this study, of which many were engorged, points to their potential role as vectors of different diseases.

The second most common species encountered in most of our collections was *Culicoides punctatus sensu stricto* (ss), and many of them were engorged (Tables 1 and 2). *C. pulicaris* ss. and *C. lupicaris* ss. were collected in lower numbers. Other authors have also reported the *C. pulicaris* group (which includes *C. pulicaris*, *C. lupicaris* and *C. newsteadi* besides *C. punctatus*) to be predominantly the second most common *Culicoides* species collected in Germany [5,26,30,39,43], but they did not identify the midges of this group up to the species level.

Even though a low number of *C. dewulfi* was detected, it has been incriminated as a vector of BTV and may play a relevant role for the transmission of midge-borne diseases in Germany [8].

Furthermore, in study C, when aiming to clarify the feeding preferences for midges, we collected a high number of *C. chiopterus* (15% of all midges collected) from sheep in comparison to cattle (3.4% of all collected midges). A significantly higher percentage was engorged (p = 0.048). Recent studies also demonstrated that *C. chiopterus* frequently feeds on sheep [13,24], although other studies in which the bloodmeal of UV-light trapped midges was analysed by PCR suggest that *C. chiopterus* midges might actually have a preference for cattle [23,44]. As this species has been incriminated as a potential vector of BTV [9], a study of its feeding preferences is warranted.

## Conclusions

These findings provide additional information about the behaviour of Palearctic *Culicoides* species, and contribute to a better understanding of the role that they can play in the transmission of pathogens. This study identified *Culicoides* from the Obsoletus complex (*C. obsoletus* and *C. scoticus*) as the most abundant species collected, followed by *C. punctatus*. To the best of our knowledge, *C. subfagineus* and *C. griseidorsum* are reported for the first time in Germany. When sheep and cattle were used as baits inside drop traps standing 10 m apart, high numbers of *C. chiopterus* were found to have fed on sheep, indicating that this species may have a preference for sheep, but further studies are needed to confirm this finding. *C. imicola,* a major vector of BTV in southern Europe, was not detected. Peak activity of midges was observed around sunset; a lower peak occurred at dawn. In our study cattle seem to be more attractive than sheep for midges, and the lower regions of the body are more likely to be attacked by biting midges. This may have consequences for optimizing the treatment with ecto-parasiticides for the control of biting midges.

## Abbreviations

BT: Bluetongue; BTV: Bluetongue virus; SV: Schmallenberg; spp.: Species; ss: Sensu stricto.

## Competing interests

The authors declare that they have no competing interests.

## Authors’ contributions

PHC, AN, BB and TA designed the study. TA carried out the field work, counted and performed the morphological identification of the midges, performed the molecular analyses, analysed the data and prepared the manuscript. AN supervised the field and laboratory work, analysis of the data and interpretation of results, participated in the field work and assisted in drafting the manuscript. WW participated in the field work and in the identifications. BB assisted in drafting the manuscript. XA assisted in the *Culicoides* identification. PHC supervised the study, and assisted in drafting the manuscript. All authors read and approved the final version of the manuscript.

## References

[B1] Mellor PS Boorman J Baylis M *Culicoides* biting midges: their role as arbovirus vectors Annu Rev Entomol 2000 45 307 340 10.1146/annurev.ento.45.1.307 10761580

[B2] Rasmussen LD Kristensen B Kirkeby C Rasmussen TB Belsham GJ Bodker R Botner A Culicoids as vectors of Schmallenberg virus Emerg Infect Dis 2012 18 7 1204 1206 22709978 10.3201/eid1807.120385 PMC3376822

[B3] Wilson A Mellor P Bluetongue in Europe: vectors, epidemiology and climate change Parasitol Res 2008 103 Suppl 1 S69 77 19030888 10.1007/s00436-008-1053-x

[B4] Wilson AJ Mellor PS Bluetongue in Europe: past, present and future Philos Trans R Soc Lond B Biol Sci 2009 364 1530 2669 2681 10.1098/rstb.2009.0091 19687037 PMC2865089

[B5] Hoffmann B Bauer B Bauer C Batza HJ Beer M Clausen PH Geier M Gethmann JM Kiel E Liebisch G Monitoring of putative vectors of bluetongue virus serotype 8, Germany Emerg Infect Dis 2009 15 9 1481 1484 10.3201/eid1509.090562 19788820 PMC2819873

[B6] Savini G Goffredo M Monaco F Di Gennaro A Cafiero MA Baldi L de Santis P Meiswinkel R Caporale V Bluetongue virus isolations from midges belonging to the Obsoletus complex (Culicoides, Diptera: Ceratopogonidae) in Italy Vet Rec 2005 157 5 133 139 16055660 10.1136/vr.157.5.133

[B7] Caracappa S Torina A Guercio A Vitale F Calabro A Purpari G Ferrantelli V Vitale M Mellor PS Identification of a novel bluetongue virus vector species of *Culicoides* in Sicily Vet Rec 2003 153 3 71 74 10.1136/vr.153.3.71 12892265

[B8] Meiswinkel R van Rijn P Leijs P Goffredo M Potential new *Culicoides* vector of bluetongue virus in northern Europe Vet Rec 2007 161 16 564 565 10.1136/vr.161.16.564 17951565

[B9] Dijkstra E van der Ven IJ Meiswinkel R Holzel DR Van Rijn PA *Culicoides chiopterus* as a potential vector of bluetongue virus in Europe Vet Rec 2008 162 13 422 18375991 10.1136/vr.162.13.422-a

[B10] Carpenter S McArthur C Selby R Ward R Nolan DV Luntz AJ Dallas JF Tripet F Mellor PS Experimental infection studies of UK *Culicoides* species midges with bluetongue virus serotypes 8 and 9 Vet Rec 2008 163 20 589 592 10.1136/vr.163.20.589 19011244

[B11] Elbers AR Meiswinkel R van Weezep E van Oldruitenborgh-Oosterbaan MM S Kooi EA Schmallenberg Virus in *Culicoides* spp. biting midges, the Netherlands, 2011 Emerg Infect Dis 2013 19 1 106 109 10.3201/eid1901.121054 23260040 PMC3558002

[B12] Conraths F Peters M Beer M Schmallenberg virus, a novel orthobunyavirus infection in ruminants in Europe: Potential global impact and preventive measures N Z Vet J 2013 61 2 63 67 10.1080/00480169.2012.738403 23215779

[B13] Carpenter S Szmaragd C Barber J Labuschagne K Gubbins S Mellor P An assessment of *Culicoides* surveillance techniques in northern Europe: have we underestimated a potential bluetongue virus vector? J Appl Ecol 2008 45 4 1237 1245

[B14] Viennet E Garros C Lancelot R Allene X Gardes L Rakotoarivony I Crochet D Delecolle JC Moulia C Baldet T Assessment of vector/host contact: comparison of animal-baited traps and UV-light/suction trap for collecting *Culicoides* biting midges (Diptera: Ceratopogonidae), vectors of Orbiviruses Parasit Vector 2011 4 119 10.1186/1756-3305-4-119 PMC3145584 21707980

[B15] Hendl M Liedtke H, Marcinek J Das Klima des Norddeutschen Tieflandes Physische Geographie Deutschlands 1994 Klett-Perthes: Gotta 559

[B16] Climate diagrams Potsdam http://www.pik-potsdam.de/services/klima-wetter-potsdam

[B17] Weiher W Bauer B Mehlitz D Nijhof AM Clausen PH Taubert A, Grevelding CG, Bauer C Field evaluation of the efficacy and safety of a deltamethrin pour on formulation (Butox protect 7.5 mg/ml) for the control of *Culicoides* midges in sheep Proceedings of the DVG conference 'Aktuelle Erkenntnisse aus der Veterinärparasitologie': 8-10 July 2013; Giessen 2013 Giessen: DVG Service GmbH 158

[B18] Berman A Effects of body surface area estimates on predicted energy requirements and heat stress J Dairy Sci 2003 86 11 3605 3610 10.3168/jds.S0022-0302(03)73966-6 14672191

[B19] Murray MD Potential vectors of bluetongue in Australia Aust Vet J 1975 51 4 216 220 10.1111/j.1751-0813.1975.tb00060.x 169797

[B20] Blackwell A King FC The vertical distribution of *Culicoides impunctatus* larvae Med Vet Entomol 1997 11 1 45 48 10.1111/j.1365-2915.1997.tb00288.x 9061676

[B21] Delécolle JC Nouvelle contribution à l’étude systematique et iconographique des espèces du genre *Culicoides* (Diptera: Ceratopogonidae) du nord-est de la France PhD thesis 1985 Université Louis Pasteur de Strasbourg: UFR des Sciences de la Vie et de la Terre

[B22] Delecolle JC Ortega MD Description d’une espéce nouvelle du genre *Culicoides* originaire d’Espagne, apparentée á C. fagineus Edwards, 1939 (Diptera, Ceratopogonidae) Nouv Rev Hematol 1998 15 283 290

[B23] Garros C Gardes L Allene X Rakotoarivony I Viennet E Rossi S Balenghien T Adaptation of a species-specific multiplex PCR assay for the identification of blood meal source in *Culicoides* (Ceratopogonidae: Diptera): applications on Palaearctic biting midge species, vectors of Orbiviruses Infect Genet Evol 2011 11 5 1103 1110 10.1016/j.meegid.2011.04.002 21511056

[B24] Griffioen K van Gemst DB Pieterse MC Jacobs F Sloet van Oldruitenborgh-Oosterbaan MM *Culicoides* species associated with sheep in the Netherlands and the effect of a permethrin insecticide Vet J 2011 190 2 230 235 10.1016/j.tvjl.2010.10.016 21169040

[B25] van der Rijt R van den Boom R Jongema Y van Oldruitenborgh-Oosterbaan MM *Culicoides* species attracted to horses with and without insect hypersensitivity Vet J 2008 178 1 91 97 10.1016/j.tvjl.2007.07.005 17728164

[B26] Clausen PH Stephan A Bartsch S Jandowsky A Hoffmann-Kohler P Schein E Mehlitz D Bauer B Seasonal dynamics of biting midges (Diptera: Ceratopogonidae, Culicoides spp.) on dairy farms of Central Germany during the 2007/2008 epidemic of bluetongue Parasitol Res 2009 105 2 381 386 10.1007/s00436-009-1417-x 19333620

[B27] Stendel W Hamel HD Sieveking HU Bruhne D Analytical determination of the distribution of flumethrin on the body surface of cattle following topical pour-on application Vet Parasitol 1992 42 1–2 137 143 1615624 10.1016/0304-4017(92)90109-m

[B28] Sallovitz JM Lifschitz A Imperiale F Virkel G Lanusse C A detailed assessment of the pattern of moxidectin tissue distribution after pour-on treatment in calves J Vet Pharmacol Ther 2003 26 6 397 404 10.1046/j.0140-7783.2003.00537.x 14962050

[B29] Bauer B Jandowsky A Schein E Mehlitz D Clausen PH An appraisal of current and new techniques intended to protect bulls against *Culicoides* and other haematophagous nematocera: the case of Schmergow, Brandenburg, Germany Parasitol Res 2009 105 2 359 365 10.1007/s00436-009-1410-4 19333621

[B30] Bartsch S Bauer B Wiemann A Clausen PH Steuber S Feeding patterns of biting midges of the *Culicoides obsoletus* and *Culicoides pulicaris* groups on selected farms in Brandenburg, Germany Parasitol Res 2009 105 2 373 380 10.1007/s00436-009-1408-y 19308450

[B31] Lassen SB Nielsen SA Skovgard H Kristensen M Molecular identification of bloodmeals from biting midges (Diptera: Ceratopogonidae: Culicoides Latreille) in Denmark Parasitol Res 2011 108 4 823 829 10.1007/s00436-010-2123-4 20978788

[B32] Schumann H Bährmann R Stark A Checkliste der Dipteren Deutschlands Studia dipterologica. Suppl 2 1999 Halle: Asphyx Verlag 1 354

[B33] Greiner EC Fadok VA Rabin EB Equine *Culicoides* hypersensitivity in Florida: biting midges aspirated from horses Med Vet Entomol 1990 4 4 375 381 10.1111/j.1365-2915.1990.tb00454.x 2133005

[B34] Knols BG Mboera LE Takken W Electric nets for studying odour-mediated host-seeking behaviour of mosquitoes Med Vet Entomol 1998 12 1 116 120 10.1046/j.1365-2915.1998.00087.x 9513949

[B35] Mands V Kline DL Blackwell A *Culicoides* midge trap enhancement with animal odour baits in Scotland Med Vet Entomol 2004 18 4 336 342 10.1111/j.0269-283X.2004.00516.x 15641999

[B36] Venter GJ Labuschagne K Hermanides KG Boikanyo SN Majatladi DM Morey L Comparison of the efficiency of five suction light traps under field conditions in South Africa for the collection of *Culicoides* species Vet Parasitol 2009 166 3–4 299 307 19758757 10.1016/j.vetpar.2009.08.020

[B37] Nelson RL Bellamy RE Patterns of flight activity of *Culicoides variipennis* (Coquillett) (Diptera: Ceratopogonidae) J Med Entomol 1971 8 3 283 291 5118869 10.1093/jmedent/8.3.283

[B38] Meiswinkel R Baldet T de Deken R Takken W Delecolle JC Mellor PS The 2006 outbreak of bluetongue in northern Europe–the entomological perspective Prev Vet Med 2008 87 1–2 55 63 18640734 10.1016/j.prevetmed.2008.06.005

[B39] Mehlhorn H Walldorf V Klimpel S Schaub G Kiel E Focke R Liebisch G Liebisch A Werner D Bauer C Bluetongue disease in Germany (2007–2008): monitoring of entomological aspects Parasitol Res 2009 105 2 313 319 10.1007/s00436-009-1416-y 19322587

[B40] Mehlhorn H Walldorf V Klimpel S Schmahl G Outbreak of bluetongue disease (BTD) in Germany and the danger for Europe Parasitol Res 2008 103 Suppl 1 S79 86 19030889 10.1007/s00436-008-1100-7

[B41] Nielsen SA Nielsen BO Chirico J Monitoring of biting midges (Diptera: Ceratopogonidae: Culicoides Latreille) on farms in Sweden during the emergence of the 2008 epidemic of bluetongue Parasitol Res 2010 106 5 1197 1203 10.1007/s00436-010-1791-4 20174825

[B42] Meiswinkel R Goffredo M Dijkstra EG van der Ven IJ Baldet T Elbers A Endophily in *Culicoides* associated with BTV-infected cattle in the province of Limburg, south-eastern Netherlands, 2006 Prev Vet Med 2008 87 1–2 182 195 18672304 10.1016/j.prevetmed.2008.06.008

[B43] Mehlhorn H Walldorf V Klimpel S Schmahl G Al-Quraishy S Walldorf U Mehlhorn B Batza HJ Entomological survey on vectors of Bluetongue virus in Northrhine-Westfalia (Germany) during 2007 and 2008 Parasitol Res 2009 105 2 321 329 10.1007/s00436-009-1413-1 19330354

[B44] Ninio C Augot D Delecolle JC Dufour B Depaquit J Contribution to the knowledge of *Culicoides* (Diptera: Ceratopogonidae) host preferences in France Parasitol Res 2011 108 3 657 663 10.1007/s00436-010-2110-9 20967462

